# A silver bullet in a golden age of functional genomics: the impact of *Agrobacterium*-mediated transformation of fungi

**DOI:** 10.1186/s40694-017-0035-0

**Published:** 2017-09-26

**Authors:** Alexander Idnurm, Andy M. Bailey, Timothy C. Cairns, Candace E. Elliott, Gary D. Foster, Giuseppe Ianiri, Junhyun Jeon

**Affiliations:** 1 School of BioSciences, University of Melbourne, Melbourne, VIC 3010 Australia; 2 School of Biological Sciences, University of Bristol, Bristol, UK; 3 Department of Applied and Molecular Microbiology, Technische Universität Berlin, Berlin, Germany; 4 Department of Molecular Genetics and Microbiology, Duke University Medical Center, Durham, USA; 5 College of Life and Applied Sciences, Yeungnam University, Gyeongsan, South Korea

**Keywords:** Functional genomics, Mycota, Pathogenicity genes, *Rhizobium radiobacter*, Transfer DNA

## Abstract

The implementation of *Agrobacterium tumefaciens* as a transformation tool revolutionized approaches to discover and understand gene functions in a large number of fungal species. *A. tumefaciens* mediated transformation (*At*MT) is one of the most transformative technologies for research on fungi developed in the last 20 years, a development arguably only surpassed by the impact of genomics. *At*MT has been widely applied in forward genetics, whereby generation of strain libraries using random T-DNA insertional mutagenesis, combined with phenotypic screening, has enabled the genetic basis of many processes to be elucidated. Alternatively, *At*MT has been fundamental for reverse genetics, where mutant isolates are generated with targeted gene deletions or disruptions, enabling gene functional roles to be determined. When combined with concomitant advances in genomics, both forward and reverse approaches using *At*MT have enabled complex fungal phenotypes to be dissected at the molecular and genetic level. Additionally, in several cases *At*MT has paved the way for the development of new species to act as models for specific areas of fungal biology, particularly in plant pathogenic ascomycetes and in a number of basidiomycete species. Despite its impact, the implementation of *At*MT has been uneven in the fungi. This review provides insight into the dynamics of expansion of new research tools into a large research community and across multiple organisms. As such, *At*MT in the fungi, beyond the demonstrated and continuing power for gene discovery and as a facile transformation tool, provides a model to understand how other technologies that are just being pioneered, e.g. CRISPR/Cas, may play roles in fungi and other eukaryotic species.

## Background

In 1998, Dunn-Coleman and Wang published a commentary on a newly described system for the transformation of foreign DNA into filamentous fungi using *Agrobacterium tumefaciens*: the catchy term in their article’s title was that this method was potentially a “silver bullet” [1]. We think of a “silver bullet” as a missile to combat werewolves or other fantasy monsters, yet here there is an additional metaphor; DNA is shot into a fungal genome to cause damage to a key gene, and thereby that can provide information on the strengths and weaknesses of the fungus. This remarkable use of a plant pathogenic bacterium, *A. tumefaciens*, to transform fungi had first been demonstrated in the model yeast *Saccharomyces cerevisiae* just a few years earlier [2, 3], and then extended in 1998 to seven species of filamentous fungi in both the Ascomycota and Basidiomycota lineages [4]. Within a decade from its first reported use in *S. cerevisiae*, by 2005 over 50 fungal species had been transformed with *A. tumefaciens* [5]. In the decade since then, the use of *A. tumefaciens* mediated transformation (*At*MT) continued to expand to become a standard experimental technique within the tool-box for gene manipulation in many fungal species. For some species it became the easiest or even the only method by which to introduce foreign DNA. In other species, it emerged as a powerful technique for forward genetics, for use in the creation of large collections of strains carrying random T-DNA insertions and their analysis, for reverse genetics to create specific targeted gene replacements, or for manipulation of gene expression for biotechnological benefits.

In this review we describe the rise and influence of *At*MT on the understanding of fundamental aspects of fungi. We describe species or groups of fungi in which *At*MT has had greatest impact, some of the limitations that have subsequently emerged in applications, and areas of research or fungal species in which this transformation technology did not have as great an impact. Understanding how this technology was implemented can guide or anticipate the benefits of future technologies for advancing research on fungi.

It is not possible to include specific details from all the publications reporting the use of *At*MT on fungi, even if covering those since the review by Michielse et al. [5]. A PubMed search of “Agrobacterium and fungus” returns more than 900 papers, and as an example in the *Cryptococcus neoformans* species complex alone *At*MT has been used in more than 30 studies. Other comprehensive and insightful reviews address specific aspects of this technique, e.g. different vectors that are available [6], or the proteins encoded by *A. tumefaciens* that are required to transform organisms [7, 8], which this review aims to complement. Finally, “impact” is relative in that what may appear important to one set of researchers may not to another set, while individual people may have personal favorite experiments or discoveries made using the technique.

## *Agrobacterium tumefaciens* and how it transforms species

*Agrobacterium tumefaciens* is a plant pathogen in the class α-Proteobacterium that is best known as one of nature’s natural agents in creating genetically modified organisms. In this process, the bacterium inserts a piece of a plasmid into the nucleus of the plant host cell, and in the wild that bacterial DNA encodes proteins that modify the plant growth in favor of the bacterium. In most circumstances, this results in the formation of a non-proliferative gall or tumor-like growth on a plant, with alterations in the recipient genome that are not normally carried on into subsequent plant generations. However, analysis of the genome of sweet potato indicates that in rare cases these transformation events can be integrated more permanently into the genome [9]. The *Agrobacterium* genus is within the family Rhizobiaceae and as such it is closely related to the genus *Rhizobium*, members of which also form intimate associations with plants to fix atmospheric nitrogen. *A. tumefaciens* was renamed *Rhizobium radiobacter* [10], although the community using this species as a transformation technology for fungi continues to use the name *A. tumefaciens*.

Before the development of genome sequencing projects, the only known example of horizontal gene transfer from bacteria to eukaryotes was the trans-conjugation mediated by *A. tumefaciens* [11]. *Agrobacterium* naturally exists in an environment where it encounters numerous hosts, including fungi that are likely to be present at the plant wounds, which induce T-DNA transfer. Knight et al. [12] demonstrated that it is entirely feasible that such transformation events happen in a natural environment. They co-cultivated the plant pathogenic fungus *Verticillium albo*-*atrum* on plant material alongside an *Agrobacterium* strain containing a plasmid that could potentially transform fungi, and observed transformation of the fungus under these *in planta* conditions [12]. Of course, in the wild such an event is unlikely to deliver any beneficial DNA sequence into the fungus, so may well not confer any selectable advantage, but it is interesting to speculate on the frequency of such events over an evolutionary timescale: indeed, in addition to plants [9], genome sequencing projects have identified *Agrobacterium*-like DNA in the genomes of some fungi such as *Aspergillus oryzae* [13].

**Fig. 1:**
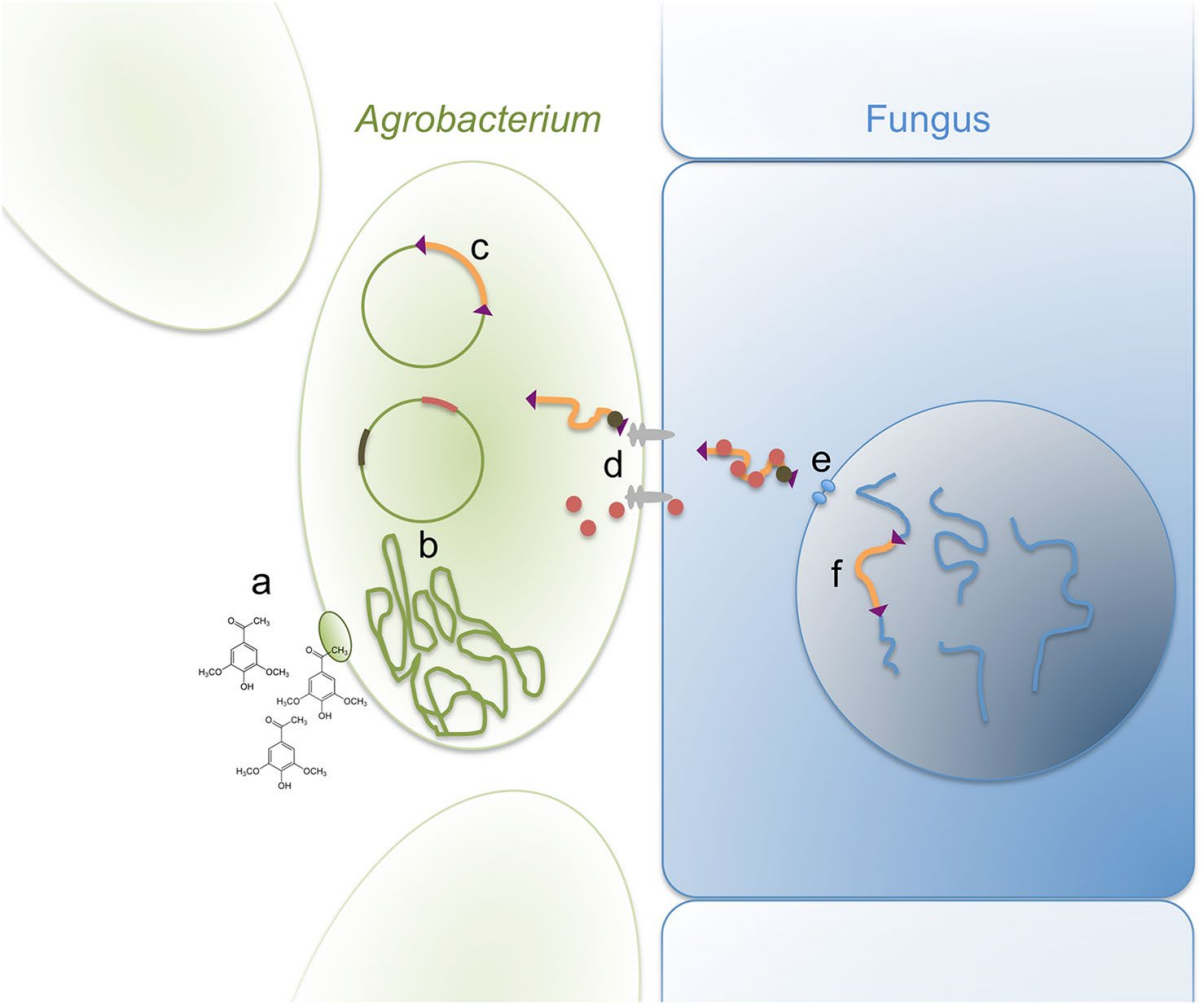
A summary of the transformation process of fungi by *Agrobacterium tumefaciens*. At step *a*, acetosyringone present in the transformation media is recognized by the bacterium, triggering *b* the expression of genes within the chromosomal DNA and from the helper plasmid. At stage *c*, the T-DNA is excised from the T-plasmid. The linear DNA is bound by VirD2, which is recognised by the type IV secretion system for export from the cell, along with other Vir proteins (*d*). At step *e*, the DNA enters the fungal nucleus guided by nuclear localization signals on the bacterial-encoded proteins. In the final event *f*, the T-DNA inserts into the chromosomal DNA of the fungus. The T-DNA should encode a selectable marker such that the integration events can be selected

Plant molecular biologists altered wild strains of *A. tumefaciens* to their own advantage. The bacterial strains and genetic material were modified to prevent gall formation, and to establish systems in which DNA for transformation into a plant can be placed between two direct repeats of 25 bp (the left and right borders of the transfer or T-DNA) (Fig. 1). From the perspective of bacterial genetics, rather than transformation it is more accurate to describe the movement of the T-DNA from the bacterium into the eukaryotic host as a trans-conjugation method of gene transfer, in this case a conjugation mechanism that is capable of occurring across different species. The promiscuous nature of *A. tumefaciens*, to target a wide diversity of hosts, enabled it to be applied to numerous other eukaryotic species, with members of the fungi being the best examples beyond the model plant *Arabidopsis thaliana* [11].

One of the initial downsides of transformation of fungi with *Agrobacterium* was the perceived inflexibility with the vector systems. Some of the *Agrobacterium* vectors were rather unwieldy, with limited restriction sites for conventional cloning and, having been developed for plants, were prefabricated with selection cassettes and regulatory elements specifically for plant transformation. Fortunately some of the suites of vectors made for plants (e.g. pGreen, pCambia, etc.) also included small vectors that had empty T-DNA regions that were amenable to easy manipulation. These have been updated to allow vector construction by means such as yeast recombination in *S. cerevisiae* [14–16], the Gateway system [17] or Golden Gate assembly [18], making vector construction and deployment simple and amenable to high throughput approaches.

Another limit of the early vectors was the length of DNA that could be inserted, which was a significant technical restriction for complementation of large genes. One solution to this problem was the development of the BIBAC system, which enabled the modification of any bacterial artificial chromosome (BAC) containing a large piece of fungal DNA to contain left and right border sequences, thus enabling direct fungal transformation. Large fragments (up to 75 kb) of DNA were successfully transferred into the *Fusarium oxysporum* f. sp. *lycopersici* genome using this method [19]. In a similar manner, a system to convert BACs into vectors suitable for *At*MT in the *Ustilago maydis* has also been developed [20].

## Advantages of *At*MT over other transformation techniques

Transformation tools existed for fungi prior to the development of *At*MT, for example using the protoplast/polyethylene glycol or cation/polyethylene glycol approaches [21, 22]. However, *At*MT provided improvements over many of these methods, explaining why it became a transformation tool of choice in many fungal species.

Firstly, *At*MT eliminates the need to remove the fungal cell wall to make protoplasts. While protoplasting is an established method in some species, in others it is difficult and variable in success. Fungi have a suite of cell wall types that differ between species and that change at different stages during growth and development. These differences likely explain why the ease and success of protoplasting vary between species; this is not helped by the difficulties in obtaining suitable cell wall degrading enzymes. In contrast, although *Agrobacterium* does show cell type preferences, as discussed later in the section on the mushroom-forming Agaricomycotina, *Agrobacterium* can transform species across a wide spectrum of evolution, including mammalian cells [23] and oomycetes [24], and many different tissue or cell types in fungi.

A second significant advantage to *At*MT over other approaches is that the T-DNA can integrate randomly into the genome. Consequently, much of the impact of *At*MT comes from the perspective of random mutagenesis as a resource for forward genetic screens. At the time of development of *At*MT, the process of restriction enzyme mediated integration (REMI) was the insertional mutagenesis method of choice. This method includes restriction enzymes in the stage when DNA is transformed into protoplasted cells. A number of problems arose with this method such as mutations not linked to the inserted DNA, which were proposed to be caused by the restriction enzymes causing damage to the DNA. Other insertional mutagenesis tools include transposon insertions, although these usually require the design of specific constructs for each species. *At*MT largely superseded REMI as the insertional mutagenesis tool in fungi [5]. Usually the T-DNA inserts as a single copy into the genome, so any change in phenotype is likely caused by the insertion. After screening libraries of T-DNA insertion transformants for phenotypes, either side of the T-DNA insert are then obtained by difference methods, which are most often PCR-based, in order to identify the affected gene. Typically, the function of the genes identified using T-DNA mutagenesis is confirmed through (a) linkage analysis of the progeny obtained from crosses between a strain of opposite mating type and the T-DNA mutant, if the mutation does not affect the sexual cycle; (b) generation of a targeted replacement allele by means of transformation techniques suitable for the studied organism, or (c) complementation with a wild type copy of the gene.

Thirdly, *At*MT is amenable for use in reverse genetic approaches for targeted gene deletion or disruption. This differs from random insertional mutagenesis, as transformation vectors are supplemented with DNA sequences that mediate homologous recombination of the exogenous cassette with specific loci of the recipient genome. Thus, *At*MT can be used for targeted replacement at desired genomic regions, most obviously a putative open reading frame. *At*MT was often developed in conjuncture with the isolation of mutants in the non-homologous end joining DNA repair process [25]. Mutation of this pathway helps increase the proportion of transformants that have gene replacements. Over the past decade, there has been a rapid increase in publicly available genome sequences of fungi [26], which has enabled facile identification of individual genes or gene families that can be analyzed by targeted gene deletion. This has occurred in parallel with development of numerous molecular tools, including inducible promoter systems [27], recyclable markers [25] and most recently CRISPR-Cas genome editing [28]. These techniques now promise functional genomic analyses at a high throughput level, and systems-level insight into industrial tractability, processes essential for disease, and putative drug targets of many fungi [29]. Consequently, targeted manipulation of fungal genomes using *At*MT is a critical technique that will facilitate the implementation of more recent breakthroughs.

Finally, having a method for easy transformation “leveled the playing field” for discovering gene function in what had up until then been dominated by the model species for molecular biology experiments. This particularly became the case for non-conventional species as soon as their genome sequence became available. Examples are given later in this review.

## Trends in the research of fungi using *At*MT

In their 2005 review, Michielse et al. [5] described specific features about *At*MT, and then some research trends many that continued over the following decade. A large focus has been on the efficiency of transformation as influenced by co-culture conditions, e.g. bacterial and fungal cell concentrations, temperature, length of co-incubation, and concentration of acetosyringone, which is a plant metabolite released from wounded roots that enhances *A. tumefaciens* transformation (Fig. 1). The direction of this focus on transformation efficiency relates to the prior challenges in obtaining large numbers of transformants from protoplasts or other methods, and hence the efforts to optimize conditions to maximize the number of transformants obtained per experiment. However, this is not crucial because *At*MT is technically easy, and if more transformants are needed they can be obtained just by increasing the number of transformation experiments to be performed. Conversely, it is ideal to generate a library of single T-DNA insertion mutants quickly and unequivocally link the phenotype of interest with the genetic mutation, but little work has addressed if the numbers of transformants obtained correlate with the number of integration events per transformant. There is a tendency for more publications reporting the first use of *At*MT in a species, and fewer on the full implementation of the technique for making mutations in genes, or other purposes. Given that the following examples represent only a small proportion of all species successfully transformed using *Agrobacterium*, there remains a large untapped resource waiting for gene discovery in a wide diversity of fungi.

## Fungal species and biological questions in which *At*MT made greatest contributions

**Fig. 2:**
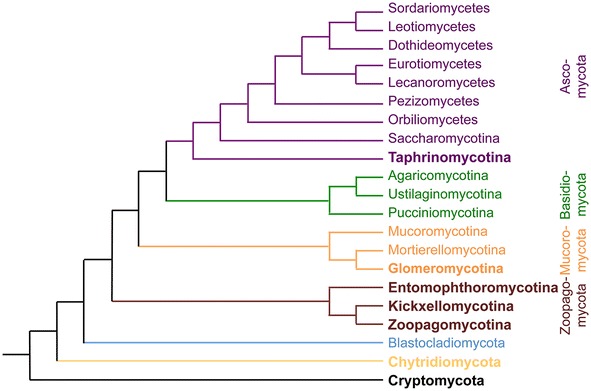
Phylogeny of major lineages within the fungi. The relationships are based on references [204, 243]; note that some nodes remain poorly defined. For hierarchy: phylum -mycota, subphylum -mycotina, class -mycetes. The Pezizomycotina subphylum is split into seven classes in the phylogeny. Groups in which species have not yet been transformed successfully by *Agrobacterium* are in bold

Application of *At*MT depends on the question being asked, and this versatile tool has suited answering such questions in different fungi. Approaches include individual gene deletion or disruption experiments, analyses of gene classes resulting in several dozen mutant strains, to the generation of large libraries consisting of thousands of strains. Nevertheless, it is notable that some species, or indeed fungal lineages, have more widely adapted *At*MT as a common research tool. The following sections are divided based on the evolution of the fungi (Fig. 2): examples from Ascomycetes with the focus on the plant pathogens, Basidiomycetes, and a brief section on the earlier, paraphyletic fungal lineages.

### Phylum Ascomycota and the role of *At*MT on understanding gene functions in plant pathogenicity

The impact of *At*MT in the ascomycetes has been of greatest relevance to the plant pathogens, with little impact on model species like *S. cerevisiae*, *Neurospora crassa* or *Aspergillus nidulans* wherein a long history of research and efficient methods for transformation, classical genetics, and gene identification were already in place. There are thousands of ascomycete species that infect plants. One way to measure the impact of *At*MT is to examine its role in understanding gene functions in particularly problematic species. In a proposed “top ten” list of plant pathogens (Table 1, [30]), many species benefited from this technique. For those species that did not benefit, this was either because efficient methods for transformation and gene discovery were already available for them (e.g. the basidiomycete *U. maydis*) or because they are obligate pathogens and therefore difficult to co-culture with *Agrobacterium* (e.g. *Puccinia* species, *Blumeria graminis* and *Melampsora lini*). In the diverse species in which *At*MT was adopted, this technology opened the opportunity for high throughput mutant screens or construction of mutant libraries. Thus, *At*MT has been applied to a number of plant pathogenic fungi including many of the economically important pathogens. Although T-DNA integration varies depending on the system, *At*MT has been consistently an efficient tool for the genetic study of fungal pathogenesis. The following sections describe ascomycete plant pathogenic species or genera in which *At*MT has been widely used, and finishes with one human pathogen example.

**Table 1:** A “top ten” list of fungal plant pathogens for research at the molecular biology level [30], and the impact of *At*MT on these species

Rank	Name	Growth capabilities	*At*MT success	Impact	Key references
1	*Pyricularia oryzae* (*Magnaporthe oryzae*)	In vitro	Yes	Major	[15, 38–42]
2	*Botrytis cinerea*	In vitro	Yes	Modest	[244]
3	*Puccinia* spp.	Obligate pathogen	No	N/A	N/A
4	*Fusarium graminearum*	In vitro	Yes	Minor	[65]
5	*Fusarium oxysporum*	In vitro	Yes	Major	[75, 76]
6	*Blumeria graminis*	Obligate pathogen	No	N/A	N/A
7	*Zymoseptoria tritici* (*Mycosphaerella graminicola*)	In vitro	Yes	Major	[52–54, 245]
8	*Colletotrichum* spp.	In vitro	Yes	Major	[84, 86]
9	*Ustilago maydis*	In vitro	Yes	Minor	[198]
10	*Melampsora lini*	Obligate pathogen	Yes	Minor	[176]

#### *Pyricularia oryzae*

*Pyricularia oryzae* (Sordariomycetes) is the causal agent of rice blast, the most serious disease of cultivated rice. The species is also referred to as *Magnaporthe oryzae* or *M. grisea* in older literature [31]. Rice blast disease destroys enough rice to feed 60 million people every year [32]. Considering that rice is a staple food accounting for major caloric and protein intake in many countries (http://www.irri.org/), the disease is one of the major threats to global food security. Due to the experimental tractability of *P. oryzae* and the socioeconomic impact of rice blast, the fungus has served as an important model to understand the biology of fungal plant pathogens [33, 34]. Although genome sequence information had offered great opportunities to discern possible genetic attributes that confer pathogenicity on the fungus [35], low efficiency of gene knockout hampered translating genome sequences into meaningful biological information. To overcome this bottleneck, insertional mutagenesis techniques such as restriction enzyme-mediated integration (REMI) and transposon-arrayed gene knockout (TAGKO) were developed to generate mutants and examine the function of disrupted genes [36, 37].

The first demonstration of *At*MT for large-scale analysis of gene functions in any plant pathogenic fungus came from a study in which over 20,000 *P. oryzae* insertional transformants were generated [38]. Southern blot analysis revealed that >80% of transformants had a single T-DNA copy within their genome. In parallel, laboratories in the USA [39] and China produced more than 150,000 *At*MT mutants, establishing the most extensive insertional mutant libraries in any plant pathogenic fungus.

**Fig. 3:**
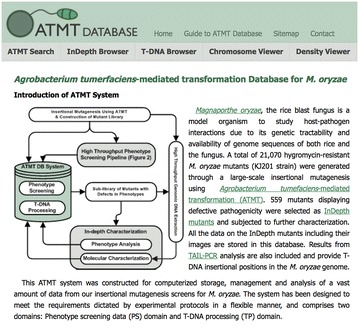
Databases of T-DNA insertion sites, mutant phenotypes, and strain availability have been developed for some fungal species. A screen shot of the front page of the ATMT Database (http://atmt.snu.ac.kr) that features information on T-DNA mutants of *P. oryzae*, primarily based on information from [38]. Limited funding for maintaining such databases and strain resources after projects end may undermine the potential impact of large-scale mutant screens

Analyses of T-DNA insertion patterns in the *P. oryzae* transformants showed that T-DNA integration favored promoter regions of genes that have an AT-rich base composition [40–42]. In addition, direct or inverted repeats of T-DNA, chromosomal rearrangements and inclusion of additional plasmid vector were also observed. Despite these biases, T-DNA insertions are relatively evenly distributed throughout all of the chromosomes, suggesting the potential of *At*MT as a tool for forward genetics. A high throughput phenotype screening system was developed to identify and characterize the transformants that are affected in key developmental steps of the life cycle, including pathogenicity [38]. The screens yielded more than 180,000 data points, which are archived and analyzed by a relational database (http://atmt.riceblast.snu.ac.kr/) (Fig. 3). Such high throughput phenotype screening in combination with identification of genes tagged by T-DNA in individual mutants led to the identification of 203 independent loci implicated in fungal pathogenicity. This represents the largest, unbiased set of putative pathogenicity genes for a single fungal species. The majority of putative pathogenicity genes tagged by T-DNA in the study included novel genes, although the list contained known pathogenicity genes, such as *NTH1*, which encodes neutral trehalase [43]. The value of the *At*MT approach is exemplified by many subsequent discoveries of genes that control aspects of fungal pathogenicity. One example amongst many is the detailed analysis of a novel gene, *DES1* (plant defense suppression 1), required for the suppression of the basal defense responses in the host plant [44]. *DES1* was identified from a T-DNA insertion site that was 750 bp from the closest predicted gene, and analysis of progeny of sexual crosses confirmed the segregation of antibiotic resistance with this interesting phenotype of aberrant conidial morphogenesis. This is an example of a gene that would not have been prioritized for analysis via a reverse genetics approach as it encodes a serine rich protein with no obvious functional domains or characterized homologues in other ascomycetes.

*At*MT can also be used to make gene replacement mutant strains in *P. oryzae*. For example, binary plasmids have been generated that are compatible with replication in *Agrobacterium* and *S. cerevisiae*, thus enabling assembly of transformation vectors using yeast recombinational cloning [15]. Such vectors can be used to create targeted gene knockouts in *P. oryzae* by homologous integration: for example, a library of 102 deletion strains in genes encoding Zn_2_Cys_6_-type transcription factors was created, with phenotypic analysis defining 61 regulators of development and eight required for infection of rice and barley [15].

Thus, for *P. oryzae*, *At*MT has had two directions of impact for gene function discovery. One was through forward genetics by screening tens of thousands of T-DNA insertional mutants. The second was in creating gene replacement strains, which were then tested for pathogenicity on plants.

#### *Zymoseptoria tritici*

*Zymoseptoria tritici* (previously named *Mycosphaerella graminicola*; Dothideomycetes) causes the most important foliar disease of wheat, Septoria blotch, with average yield losses of 5–10% annually [45]. Throughout Europe, the crop destruction and deployment of antifungals to combat *Z. tritici* are estimated to run into billions of Euros, with losses in Germany, for example, estimated to cost 500 and 310 million per year, respectively [45]. Worryingly, resistance of *Z. tritici* to every class of antifungal compounds is increasing, yet the number of new compounds developed to combat this pathogen is in decline [46]. Consequently, validation of new pathogen targets for rational fungicide development, concomitant with development of highly resistant wheat varieties, are pressing objectives for food security. *At*MT has been essential for improved understanding of the molecular basis of *Z. tritici* infection, which may ultimately lead to durable disease control strategies.

The first transformation of *Z. tritici* utilized a classical protoplast-polyethylene glycol (PEG) approach, whereby the cell walls of in vitro grown conidia were digested and a DNA cassette encoding hygromycin or carbendazim resistance was introduced into recipient genomes [47]. From this study, several limitations to protoplast-based transformation became apparent. Firstly, there were significant variations in successful protoplast generation between *Z. tritici* isolates. Secondly, this experimental challenge was further complicated by the commercial discontinuation of Novozyme 234, which was the enzyme mix used to digest conidial cell walls [47]. While other enzyme preparations exist for fungal protoplasting [48], variations in availability of validated transformation reagents introduced an additional challenge for the widespread adoption of this approach by the *Z. tritici* research community.

Consequently, with few exceptions [47, 49, 50], molecular studies of *Z. tritici* rely on *At*MT [51]. As one example, a T-DNA mutagenesis forward genetic screen generated an insertional library of 615 *Z. tritici* transformants [52]. Virulence analysis of this mutant library revealed one isolate, 5-29H, which was avirulent in a detached leaf infection assay. Mapping of the T-DNA insertion locus revealed disruption of a putative mannosyltransferase-encoding gene, and subsequent phenotypic screening and proteomic analyses demonstrated that protein *N*-glycosylation was essential for a switch from yeast-like conidia to infectious hyphal growth, and ultimately successful disease initiation [52]. Using *At*MT in a targeted approach, Cousin and co-workers deleted a mitogen-activated protein kinase (MAPK) encoding gene that had high sequence homology to the *FUS3* gene of *S. cerevisiae* [53]. This MAPK plays a critical role in mating and growth in this yeast, and deletion in *Z. tritici* resulted in aberrant polarized growth and deficiencies in host penetration during infection. In a similar approach using *At*MT, deletion of a gene encoding another MAPK, termed MgSlt2, demonstrated a critical role of this gene in resistance to several fungicides, and hyphal branching following leaf penetration [54]. Taken together, these studies demonstrate that impaired hyphal development, deficient stomatal penetration [53], or an inability to proliferate after disease initiation [54] all result in reduced pathogenicity, and ultimately suggest interference with the normal *Z. tritici* developmental program offers an opportunity to inhibit disease. Thus, *At*MT represents a critical tool in the researchers’ repertoire for identifying the targets for the rational development of novel fungicides. With regards to future *Z. tritici* experiments, and the molecular analyses of phytopathogenic fungi in general, it is clear that *At*MT will continue to be utilized even in face of major paradigm shifts in the field of fungal pathogenicity.

#### *Fusarium* species

Members of the *Fusarium* genus (Sordariomycetes) include agronomically important plant pathogens some of which are known for producing mycotoxins and also as opportunistic human pathogens [55]. One recent phylogenetic study suggests that the *Fusarium* genus could be subdivided into 20 species complexes with the terminal *Fusarium* clade originating in the middle Cretaceous period [56]. The most intensely studied *Fusarium* species include members of the species complexes Sambucinum (*F. graminearum*, *F. pseudograminearum* and *F. culmorum* causing blights and rots of wheat and barley), Fujikuroi (*F. fujikuroi* causing bakane disease of rice and *F. verticillioides* causing ear rot of maize), and Oxysporum (a species complex of soil-borne filamentous fungi whose members include more than 12 forma speciales causing vascular wilts of many different plant species). The Oxysporum complex has been a focus of interest to evolutionary biologists due to the range of life strategies of its members. Although all members are soil-borne, not all isolates are plant pathogens, a feature associated with the elevated genome plasticity of *Fusarium* and that allow the acquisition of mobile pathogenesis chromosomes [57]. Moreover, the finding that an isolate pathogenic on tomato can cause disease in immunocompromised mice raised the profile of this species complex as a model for cross-kingdom pathogenesis studies [58]. Given the size, diversity and economic damage caused by members of the genus it is not surprising that *At*MT has made an impact on some species of *Fusarium* more than others, and therefore the main focus of this section will be on a limited number of species such as *F. graminearum* and *F. oxysporum*.

In the first comprehensive report of transformation of a *Fusarium* species (*F. oxysporum* f. sp. *raphani* strain 699), the efficiency ranged from 1 to 40 transformants per 10^7^ protoplasts [59]. Although transformation efficiency using protoplasting methods was low, homologous recombination was easily achieved in *F. oxysporum* and *F. graminearum* with 20–50% homologous integration events [60, 61], opening the way for reverse genetic experiments. The development of *At*MT for filamentous fungi including *F. venenatum*, the source of the edible mycoprotein Quorn™, promised to improve transformation efficiency for the genus *Fusarium* [4]. Two more *Fusarium* species, *F. circinatum* and *F. oxysporum*, were transformed in 2001, providing new vectors for fungal transformation as well as a plasmid rescue cassette to enable the easy retrieval of DNA sequences flanking the T-DNA insertion in the fungal genome [62, 63]. These publications reported improved transformation efficiencies to 300–500 transformants per 10^6^ conidia thus paving the way for further forward genetic approaches in *Fusarium* [64]. Since 2001, *At*MT has been reported in at least nine *Fusarium* species including *F. culmorum*, *F. graminearum*, *F. pseudograminearum* [65], *F. verticillioides* [66], *F. virguliforme* [67], and *F. avenaceum* [68].

Although *F. graminearum* can be transformed by *A. tumefaciens*, the efficiency of *At*MT using non-homologous DNA is extremely low. Malz et al. [65] compared the efficiency of *At*MT for the random integration of a hygromycin resistance cassette into three species of *Fusarium* and found that in a single transformation experiment *F. pseudograminearum* yielded 409 transformants, whereas *F. culmorum* and *F. graminearum* yielded only 13 and 9 transformants, respectively. Interestingly, *F. graminearum* can be transformed to high efficiency (up to 2000 transformants per 10^7^ conidia) with *At*MT as long as the vector used contains some homologous *F. graminearum* DNA within it, suggesting that homologous recombination may be the dominant type of integration event in *F. graminearum* as it is in *S. cerevisiae* [69]. Consequently REMI, and not *At*MT, is still used for forward genetics approaches in *F. graminearum* [70].

In contrast, *At*MT is clearly the method of choice for large-scale random mutagenesis approaches in *F. oxysporum.* The first random mutagenesis studies published were done using REMI [71, 72] or transposon tagging [73, 74] with numbers of mutants generated in the range of 182–1129. *At*MT has enabled studies of a much larger scale. In 2009, two large-scale studies were published using *At*MT. Screening of a mutant collection of 10,290 transformants of *F. oxysporum* f. sp. *lycopersici* identified 106 isolates with reduced pathogenicity on tomato and 111 potential pathogenicity genes [75]. Similarly, Li et al. [76] created a bank of 20,000 mutants of *F. oxysporum* f. sp. *cubense* race 4, and screened them over a 6-year period for reduced pathogenicity on Cavendish banana plantlets. This revealed 27 reduced or loss of pathogenicity isolates, one of which had a T-DNA insertion in the gene *FoOCH1* that encodes an α-1-6-mannosyltransferase. Interestingly, further characterization of *FoOCH1* via targeted deletion and complementation experiments was achieved by transforming protoplasts, illustrating that in contrast with fungal systems covered in other sections of this review, there is a choice of efficient transformation methods available to researchers working on *F. oxysporum*.

#### *Colletotrichum* species

The genus *Colletotrichum* (Sordariomycetes) contains a large number of plant pathogens causing diseases in most crops, including grains and fruit, as well as resulting in post-harvest losses [30, 77, 78]. Many species are hemibiotrophs (i.e. growing first as a biotroph without causing disease symptoms before switching to a necrotrophic mode of damage), although some purely nectrotophic species are also known.

Early insertional mutagenesis approaches in *Colletotrichum* spp. yielded discoveries into genes required for pathogenicity, indicating that insertional mutagenesis would be an effective approach for gene discovery. Examples of successful gene identification include those encoding a class V chitin synthase in *C. graminicola* [79] and a serine/threonine protein kinase in *C. lindemuthianum* [80]. However, large numbers of transformants have been made using *At*MT and screened on plants.

The current “record holders” in terms of strain numbers are *C. higginsianum*, a pathogen of Cruciferae species, and *C. gloeosporioides*, a pathogen with a wide host range that includes both monocot and dicot plants. For *C. higginsianum* three sets of mutants that total more than 21,000 T-DNA mutants have been isolated and screened on *A. thaliana* [81–84]. These studies both traced the stages during the infection cycle in which mutants were blocked in causing disease, and went on to identify 17 new pathogenicity genes for this species. For *C. gloeosporioides* two screens on more than 14,000 T-DNA strains in total have been conducted [85, 86]. The recent study by Wu et al. used an in vitro screen to identify genes required for the production of asexual spores that are required to establish disease to identify 11 candidates for genes required for conidiation [85]. Cai et al. generated more than 4000 insertional strains and screened them on detacted rubber tree leaves, to identify 16 genes required for pathogenicity [86]. In addition to random insertional mutagenesis, *At*MT can be used in *Colletotrichum* species to make targeted gene replacements, and this has been enhanced by the isolation of mutants in the Ku genes for the non-homologous end joining DNA repair pathway [82]. This pathway is involved in the ectopic insertion of DNA into fungal genomes, and thus pathway mutants have a high proportion of targeted gene replacement events after transformation.

An example of convergent discoveries by using *At*MT in *Colletotrichum* species has been the recent independent identification of T-DNA insertional mutants in the same signaling pathway in *C. higginsianum* [87] and *C. orbiculare* [88]. Both studies found insertions in components of the Regulation of Ace2 and Cellular Morphogenesis (RAM) pathway, a complex of two kinases and associated proteins that controls cell morphology in fungi. With six components, collectively this provides a large target for T-DNA insertions, and as discussed under the section on the basidiomycete *C. neoformans*, the same pathway was also first identified as impacting multiple functions in this human pathogenic yeast from T-DNA insertional mutants [89].

#### *Leptosphaeria maculans*

A plant pathogen that is not included in the “top 10” [30] list is *Leptosphaeria maculans* (Dothideomycetes), in which substantial use of *At*MT has been made as an insertional mutagenesis tool. *L. maculans* is a phytopathogen capable of attacking cultivated Brassicas such as *B. napus, B. rapa, B. juncea, B. oleracea* as well as numerous wild Cruciferae species [90].

*Leptosphaeria maculans* was among the first ascomycetes genetically transformed, by PEG-mediated transformation of protoplasts [91]. The first forward genetic screen in *L. maculans* was undertaken using REMI, a method that has been discussed above in sections “Advantages of *At*MT over other transformation techniques” and “*Pyricularia oryzae*”. A screen of 516 transformants identified 12 loss of pathogenicity mutants [92]. An evaluation of 47 randomly selected insertional mutants revealed complex patterns of insertions with 31 containing insertions at multiple loci, 12 with single loci insertions of multiple copies of plasmid and only 4 had single copies of the insertion plasmid, aptly illustrating the drawbacks of REMI discussed previously. PEG-mediated transformation was also used successfully to deliver gene disruption cassettes to *L. maculans* resulting in the disruption of genes, although at frequencies as low as less that 0.25% of transformants [93].

*At*MT was first shown to be effective in *L. maculans* with the successful knockout of genes encoding an ATP binding cassette transporter and two-component histidine kinase [94]. The frequency of gene deletions achieved in this study was still less than 1 in 140, therefore a negative selectable marker was developed whereby two copies of a thymidine kinase gene from the herpes simplex virus were amended to the ends of the deletion vector. Transformants arising through ectopic integration of plasmid DNA would still contain the thymidine kinase negative selectable marker and should not grow in the presence of thymidine analogues fluorodeoxyuridine or trifluorothymidine. This strategy increased the rate of homologous integration up to 1 in 30.

The greatest impacts of *At*MT in *L. maculans* are its use as a forward genetics tool for random mutagenesis and a transformation tool to deliver gene constructs that manipulate the expression of endogenous fungal genes. In comparison with other fungi discussed in this review, the forward genetics studies are smaller in scale and highlight some of the unusual events that can occur when a T-DNA is inserted into a host genome. For example, in the first forward genetic approach published, 91 transformants were screened to identify one reduced pathogenicity mutant with a T-DNA insertion in the promoter region of two divergently described genes that resulted in increased expression of both genes and rendered the transformed strain “uncomplementable” [95]. To circumvent this problem *At*MT was used to recreate this state of overexpression in new mutant strains, overexpressing both genes individually or together, and this led to the finding that overexpression of a maleylacetate reductase results in loss of pathogenicity in *L. maculans*. Interestingly this effect of T-DNA insertion altering the gene expression or transcript stability in unexpected ways has since been reported on several occasions [96, 97]. In two independent studies, the T-DNA inserted into the 3′ regulatory sequences of genes thereby altering the length and/or stability of transcripts, and resulted in the discovery of the *IFRD* gene, which is important for cell wall integrity, conidial germination and pathogenicity, and the *cpcA* gene, which is responsible for regulating the production of amino acids during starvation growth. In both of these studies where the insertion of T-DNA resulted in a complex phenotype, *At*MT was also used to deliver RNA interference constructs to create isolates with reduced expression of the gene of interest.

Blaise et al. [98] and Bourras et al. [99] published the largest and most extensively characterized forward genetic screens conducted in *L. maculans* to date. Blaise et al. found that 53 transformants out of 1388 tested had altered but reproducible pathogenicity phenotypes, ranging from lost, reduced, delayed and growth condition dependent defects. By genetic crossing of 12 mutants they could show that the T-DNA insertion was linked to the loss of pathogenicity in only 50% of the cases, thus highlighting a limitation of *At*MT. They retrieved left border sequences from 135 randomly selected transformants and observed a trend towards integration into gene rich regions with a possible bias towards regulatory or intergenic regions. These findings were substantiated in much greater detail by Bourras et al. [99] whereby 400 border sequences were obtained through thermal asymmetric interlaced (TAIL)-PCR and primer walking, thus identifying 318 single locus T-DNA integration events. With the backing of an annotated genome, the authors were able to confirm that 97% of T-DNA integrations were mapped into GC-rich and transcriptionally active regions of the genome. There was also some evidence of chromosomal bias with statistically more T-DNA insertions in chromosomes 5 and 10 and less in chromosome 18 than would have been predicted for completely random insertions. A detailed examination of insertions into the gene-rich areas showed that there were more insertions into gene regulatory regions and introns than would have been expected under a random integration hypothesis, and less insertions than expected in intergenic regions and exons. Futhermore, a comparison of the promoter sequences of targeted genes with the T-DNA left border flanking sequence revealed 5 bp long consecutive stretches of homologous sequences, termed microhomology domains, consistent with the integration of T-DNA into the *L. maculans* genome via a microhomology-mediated end-joining pathway. What is readily apparent from these studies is that T-DNA insertion into the *L. maculans* genome does not necessarily result in gene disruption or loss of gene function, and extreme truncations and chromosomal translocations can occur [100].

Given that all known genes encoding effectors, which are small secreted proteins involved in plant pathogen-host recognition, characterized in *L. maculans* to date are located in AT-rich regions of the genome and if one would assume that genes required for infection are lowly expressed in culture [101], the efficiency of *At*MT to target pathogenicity genes is questionable. However, the ease with which *L. maculans* can be transformed via *At*MT offers realms of opportunities for other forward genetics screens and the delivery of genome tailoring enzymes in the future. Furthermore, *At*MT is used to deliver RNA silencing constructs into *L. maculans*, and can be used to confirm the functions of effector genes [102].

#### *Histoplasma capsulatum*

The ascomycetes also include a number of human pathogenic species, such as *Histoplasma capsulatum* (Eurotiomycetes), the etiologic agent responsible for histoplasmosis or “cave disease”. It is an infection of the lungs normally arising through inhalation of fungal spores and is especially common in immunocompromised patients. *H. capsulatum* is a thermally dimorphic fungus characterized by two different growth forms. The saprophytic form of the fungus grows as a filamentous mold with aerial hyphae in the environment (especially in soil that contains bird or bat droppings) whence spores can become airborne and inhaled by people; the person’s body temperature allows the pathogen to grow into the next stage of its life cycle that consists of a yeast that can infect lungs, or it can travel to lymph nodes and spread through the bloodstream to other parts of the body, such as the central nervous system [103]. *H. capsulatum* can also cause significant mortality and morbidity in healthy hosts with approximately 25,000 estimated life-threatening infections per year in countries where the fungus is endemic [104].

Molecular research on *H. caspulatum* has been performed for many years, and technological developments were reviewed more than a decade ago [105]. Electroporation was the most successful technique for transformation compared to biolistic and lithium acetate/PEG-mediated transformation for efficiency and reliability, although episomal plasmids and multiple random insertions of heterologous DNA limited the exploitation for its use in functional genetics studies despite several optimization attempts [106]. As a further step toward the development of reliable and efficient molecular tools, a protocol based on *At*MT was developed for *H. capsulatum* and the related dimorphic species *Blastomyces dermatitidis* [107]; since then, *At*MT protocols have been developed for other dimorphic fungi such as *Coccidioides* spp., *Sporothrix schenkii*, *Paracoccidioides brasiliensis* and *Talaromyces* (*Penicillium*) *marneffei* (see review [108]). We focus attention on *At*MT of *H. capsulatum* and the main discoveries arising from use of this technique.

Sullivan and colleagues [107] compared the feasibility of *At*MT using two different selection markers, the native *H. capsulatum URA5* gene, and the *hph* gene (for hygromycin resistance) placed under the control of the *A. nidulans gpd* promoter and *trpC* terminator. In both *H. capsulatum* and *B. dermatitidis*, a 5- to 10-fold higher transformation efficiency was achieved using the selection for uracil prototrophs. Moreover, T-DNA insertions were always found in the host genome with more than 80% of transformants obtained bearing single T-DNA insertions; however, a small percentage of multiple copies of T-DNA, small rearrangements or deletions, and integration of plasmids regions beyond the T-DNA borders were also observed. As an insertional mutagenesis tool, *At*MT works most effectively when the DNA is transformed into uninucleate cells, which are more easily obtained for *H. capsulatum* than for *B. dermatitidis.*

Following this first report of *At*MT in these dimorphic species, other promoters (i.e. from *TEF1*), selection markers or reporter genes (i.e. *GFP, BLE*), and other transformation parameters were optimized [109]. For example, Marion et al. [110] optimized *At*MT and went on to identify *H. capsulatum* loci that impact the production of cell wall α-(1,3)-glucan, based on a simple and effective visual screening: wild-type strains of *H. capsulatum* have a visibly “rough” colony morphology on culture plates, while mutants that lack α-(1,3)-glucan have a “smooth” colony appearance. Beside *AGS1*, which was already characterized as important for cell wall construction in *H. capsulatum*, two novel genes (*AMY1* and *UGP1*) required for α-(1,3)-glucan biosynthesis were identifed, of which *AMY1* was also found to be required to kill macrophages and to colonize murine lungs [110].

The dimorphic transition is key for pathogenicity, but until 2008 little was known about what genes regulated the transition. Sil and colleagues identified *At*MT mutants that were unable to make the transition from the filamentous (fuzzy colonies) to the pathogenic yeast form (smooth colonies) under temperature shift from room temperature to 37 °C [111, 112]; the mutated genes were named *RYP1, RYP2* and *RYP3* from “*r*equired for *y*east *p*hase growth”. Subsequent studies demonstrated that these genes encode a connected network of transcription factors that regulate each other and target common genes to activate a transcriptional program that is required for cell shape changes and expression of virulence genes in response to host temperature in *H. capsulatum* [113].

*At*MT was also used to identify genes of *H. capsulatum* required for intracellular growth and virulence by assessing the survival rate of T-DNA mutants within macrophages, which led to the identification of the genes *VMA1* and *HSP82*, both crucial for virulence in an pulmonary murine model for histoplasmosis [114, 115]. More recently, in another *At*MT screen, Isaac and colleagues revealed a mechanism of evasion of *H. capsulatum* from macrophages that involves the protein calcium-binding protein Cbp1, which had been previously characterized [116] and also identified by another group using *At*MT coupled with reverse genetics and PCR screening [117]. In their screen the authors identified three independent *cbp1* mutants that grew at wild type level within macrophages but failed to elicit host-cell death; *cbp1* mutants also showed attenuated virulence in an animal model, thus suggesting a key role for Cbp1 in favoring dissemination of the fungus in the host through a mechanism that seems to be specific for *H. capsulatum* and related dimorphic fungi.

#### Other pathogenic ascomycetes

Whilst *AtMT* has had considerable success in studying gene function in plant pathogens, it has also been deployed in other pathogenic fungi, in some cases to investigate the wider functional applicability of the virulence factors first characterized in other fungal species.

Amongst insect pathogenic fungi, *Metarhizium* spp. (e.g. [118]), *Beauveria* spp. (e.g. [119]) and *Lecanicillium lecanii* [120] (all three in the Sordariomycetes) have been transformed by this method, with a sizable *At*MT T-DNA mutant collection generated in *B. bassiana* [121]. In *Metarhizium* spp, targeted gene disruption has been reported using this approach to characterize genes such as the non-ribosomal peptide synthase needed for serinocyclin synthesis [122] and further developed for high throughput gene disruption [123].

Amongst mycopathogenic fungi, *Coniothyrium minitans* (Dothideomycetes), a fungal parasite of the plant pathogen *Sclerotinia sclerotiorum*, has been successfully transformed by *At*MT [124], and in the mushroom pathogen *Lecanicillium fungicola* (Sordariomycetes), the method has been used for targeted disruption of cell wall degrading β-1-6 glucanase [125] and the Pmk1-like MAP kinase [126], with mutation of the latter gene somewhat surprisingly not having any impact on virulence.

### Phylum Basidiomycota

Basidiomycetes are distinguished morphologically by their sexual spore formation, produced on the ends of club-shaped cells (basidia) in which meiosis has taken place. The phylum is divided into three major subphyla, the Agaricomycotina, Pucciniomycotina and Ustilaginomycotina (Fig. 2). While some species of basidiomycetes (e.g. *U. maydis*, *Coprinopsis cinerea* or *C. neoformans*) have served as models for aspects of plant pathology, medical mycology, fungal or general biology, compared to ascomycetes relatively less was known about gene functions in the phylum prior to the advent of *At*MT.

It was fortuitous that the two main model basidiomycetes that had been preferred for classical Mendelian genetics, the inkcap toadstool *C. cinerea* (Agaricomycotina) and maize smut *U. maydis* (Ustilaginomycotina), both proved to be readily amenable to protoplast-based transformation methods. These species provided reliable and reproducible starting material for protoplasting, in the form of asexual ooidia for *C. cinerea* and yeast-like sporidial growth for *U. maydis*. Both species gave good yields of transformants, and *U. maydis* had the additional benefit of having both integrative and autonomous transformation vectors, and a very efficient homologous recombination system allowing easy gene targeting. The early progress achieved in these species encouraged researchers to investigate the tractability of other basidiomycetes, but difficulties were often encountered when attempting to transfer the methods developed in these models to other species. In particular the absence of asexual spores in the majority of species in the Agaricomycotina meant that protoplasting had to be performed on highly variable mycelial cultures, and the obligate pathogens such as the Pucciniomycotina species causing rusts were largely ignored due to the inherent problems in only being able to work with such species in planta.

#### The mushroom-forming species of Agaricomycetes

Historically the transformations of species in the Agaricomycetes (Agaricomycotina) were often of very low efficiency, variable in terms of success, and few suitable vectors had ever been developed. Indeed, for the cultivated button mushroom *Agaricus bisporus*, only one lab was successful in protoplast-based transformation. The report in 1998 of transformation of seven fungi [4], including *A. bisporus*, was therefore met with great excitement by the basidiomycete community and was followed by a flurry of papers on different species, although often without the high transformation frequencies seen in ascomycetes. A number of confounding factors then became apparent that help explain why the initial transformation attempts were often without success.

The key breakthrough came when Chen et al. [127] demonstrated that whilst most tissues of *A. bisporus* would only yield low transformation efficiencies, the use of gill tissue excised from fruiting bodies immediately prior to veil-break gave high efficiencies. A similar situation occurs in the important forestry pathogen *Armillaria mellea*, where the most amenable tissue for transformation is basidiospores collected from either wild-grown fruiting bodies [128] or laboratory-raised fruiting bodies [129]. These studies flag the importance of selecting the appropriate developmental stage of fungal material, since not all stages are equally amenable to transformation.

Another breakthrough in the transformation of the Agaricomycetes came with the observation that transgenes often needed to include an intron, ideally at the 5′ end of the gene. This proved to be important whether the selection or antibiotic resistance cassette was introduced via protoplasts or via *At*MT, and impacted both on choice of reporter genes and on some of the selectable markers [130]. This requirement varies from species to species, and indeed from gene to gene. For instance it is fortuitous that it is not normally required for the function of the hygromycin resistance gene typically used in initial selection of transformants, but is needed in some cases for successful deployment of the gene conferring resistance to phleomycin [131].

The choice of promoters to drive transgene expression is important because this determines when, where and if the transgene is expressed. Not all fungal promoters are active when transferred into the genome of a related species and this had to be assessed on a case-by-case basis, which added constraints to the wider utility of some of the vectors [132]. Examples from the Agaricomycetes illustrate this point. Whilst the *A. bisporus gpd* promoter showed a reasonable spectrum of activity in other fungi, there were instances where it was not very successful (e.g. [133, 134]) and there was no readily apparent pattern to explain this. This is in contrast to many of the ascomycete vectors in common use where promoters such as *trpC* or *gpdA* from *A. nidulans* have been used in other ascomycete species over wide evolutionary distances. Effective promoters that function a cross phyla are less common in Agaricomycotina, but it is perhaps noteworthy that the DNA immediately to the 5′ of the start codon of the *C. neoformans* actin gene was successful in driving hygromycin resistance in *Hypsizigus marmoreus*, *Flammulina velutipes*, and *Grifola frondosa*, suggesting that this promoter may have broad utility [135]. Curiously, RNA-seq data indicate that this DNA is a combination of promoter and the 5′ untranslated region, with that region containing an intron that is spliced in *C. neoformans* [136].

To add further complexity to the deployment of *At*MT in basidiomycetes, Kilaru et al. [131] highlighted that two slightly different forms of the hygromycin resistance cassette were in common usage, and that these gave very differing transformation efficiencies in a species-specific manner. Once all these factors—cell material, introns in markers, promoters and cassettes—were fully appreciated and factored into planned investigations, it has become far easier to transform basidiomycetes. This has allowed a wide set of studies to be undertaken on diverse aspects of basidiomycete biology.

To date, the transformation experiments on the Agaricomycetes have primarily focused on species with either edible fruiting bodies or where there is a biotechnological application, and publications have focussed on methodological development. This methodology has since been deployed to help assess expression patterns using reporter genes and now mutant screens in other species such as *Laccaria bicolor* [137]. One interesting approach has been in modifying the stress tolerance in fruiting bodies, for example in conferring cold-tolerance to the paddy straw mushroom *Volvariella volvacea* [138]. In other cases, the yield of pharmaceutically relevant compounds, such as ganodermic acid in *Ganoderma lucidum* [139], clavaric acid in *Hypholoma sublateritium* [140] or various triterpenes in *Antrodia cinnamomea* [141], has been enhanced as a result of *At*MT by overexpression of a core biosynthetic gene. One drawback to using *At*MT for overexpression is that it usually only delivers a single copy of the transformation construct and may not achieve as high a titre of the desired compound. In contrast, protoplast-mediated events often result in multi-copy integrations, delivering a wider range of expression levels, with some transformed strains having very high titre, which can be beneficial to create high expression strains.

Perhaps the most powerful application of *At*MT is in delivering constructs to effect gene silencing. This is of particular interest in basidiomycetes as the hyphae, which are often the starting material for transformation, are often maintained in a dikaryotic state, precluding the easy use of gene disruption (which requires nuclear integration of a construct) to assess gene functionality. Because post-transcriptional gene silencing operates within the cytoplasm, it has a dominant effect and thereby can cause a phenotype in the mutant lines despite their heterokaryotic state. Effective gene silencing has been deployed in a number of basidiomycetes, including the important mycorrhizal symbiont *L. bicolor* [142, 143] and in *A. bisporus* where it has been deployed to identify core synthetic genes and also the proteases involved in nutrient acquisition [144, 145].

Transformation, however, is still often challenging. While some species are naturally amenable to gene targeting/deletion via homologous recombination, and others can be made amenable by use of mutants in the non-homologous end joining pathway such as *KU70* mutants (e.g. *C. cinerea* [146]), many still have no reports of successful gene targeting. The recent high profile report of successful deployment of CRISPR/Cas in *A. bisporus* [147] may serve to overcome these issues, and we would expect the Cas proteins and guide RNA construct(s) to be deployed using *Agrobacterium*-vectors given how successful they have been in the Agaricomycetes in broadening the range of species amenable for transformation.

#### The *C. neoformans* species complex

*Cryptococcus* species are major fungal pathogens of humans within the Agaricomycotina [104], divided into serotypes, varieties, two species *C. neoformans* and *C. gattii*, and the most recent classification splitting *C. neoformans* into two species and *C. gattii* into five species [148]. All species cause disease in humans and animals, and among them, *C. neoformans* (sensu stricto) is the one most commonly isolated in clinical settings [149]. In immunocompromised individuals the fungus infects the lungs, crosses the blood–brain barrier and invades the cerebrospinal fluid, causing fatal meningitis if untreated [150]. The disease causes hundreds of thousands of deaths globally each year [151].

Molecular studies of *C. neoformans* benefit from effective tools for random and targeted mutagenesis, conditional gene expression, gene editing and protein localization. Moreover, approaches of functional genetics were dramatically streamlined by the availability of genome sequences for several *Cryptococcus* species since the early 2000s (see review [152]). Electroporation and biolistic methods were the first transformation methods developed for *C. neoformans* [153, 154] and were employed for delivering episomal plasmids into the fungus, heterologous gene expression and gene-targeted mutagenesis. As tools for random insertional mutagenesis they have been used to study several biological processes in *C. neoformans*: examples are the identification of the essential gene *CAM1* encoding calmodulin through fortuitous insertion of a marker in the 3′ UTR of the gene yielding a temperature-sensitive mutant [155], and an insertion in a chloride transporter required to balance ions for the synthesis of the virulence factor melanin [156]. Although effective as transformation tools, both electroporation and biolistics are characterized by the high rate of genetic instability of transformants probably due to transgenes not integrating into the host genome, with reports ranging from 70 to 85% of such transformant being unstable [149]. Therefore electroporation and biolistics are mainly used for the generation of targeted mutants through homologous recombination [153, 154].

*At*MT was first used for the *C. neoformans*/*C. gattii* complexes in the early 2000s [157, 158]. The selective markers used included those that confer resistance to nourseothricin, G418 and hygromycin [157, 159], which have been extensively used for *At*MT functional genetics studies. Other plasmids for *At*MT of *C. neoformans* include those enabling the fusion with genes to assess protein localization and conditional promoters. From these early reports, the high potential of *At*MT became clear when compared to electroporation and biolistic transformation, with the advantages of having a higher rate of transformation and stability of the T-DNA insertion (close to 100%). One relevant limitation or feature of *At*MT for *Cryptococcus* is that it has not yet been successfully deployed for targeted gene replacement [158], making it different from most other fungi.

*At*MT has featured in more than 30 studies on *Cryptococcus* species, many of which have used the T-DNA insertions as a mutagenic tool in forward genetics. As such, *At*MT has been valuable for the identification of *Cryptococcus* genes that are not conserved in *S. cerevisiae*, which represents a reference organism in fungi and thus a starting point for the identification of *C. neoformans* orthologs whose specific function is in general assessed by targeted mutagenesis. However, some of the most significant discoveries in *Cryptococcus* species have been made by extending the capabilities of *At*MT beyond just an insertional mutagenesis tool for wild type strains. Hence, an additional focus is placed on these species in the following sections.

**Fig. 4:**
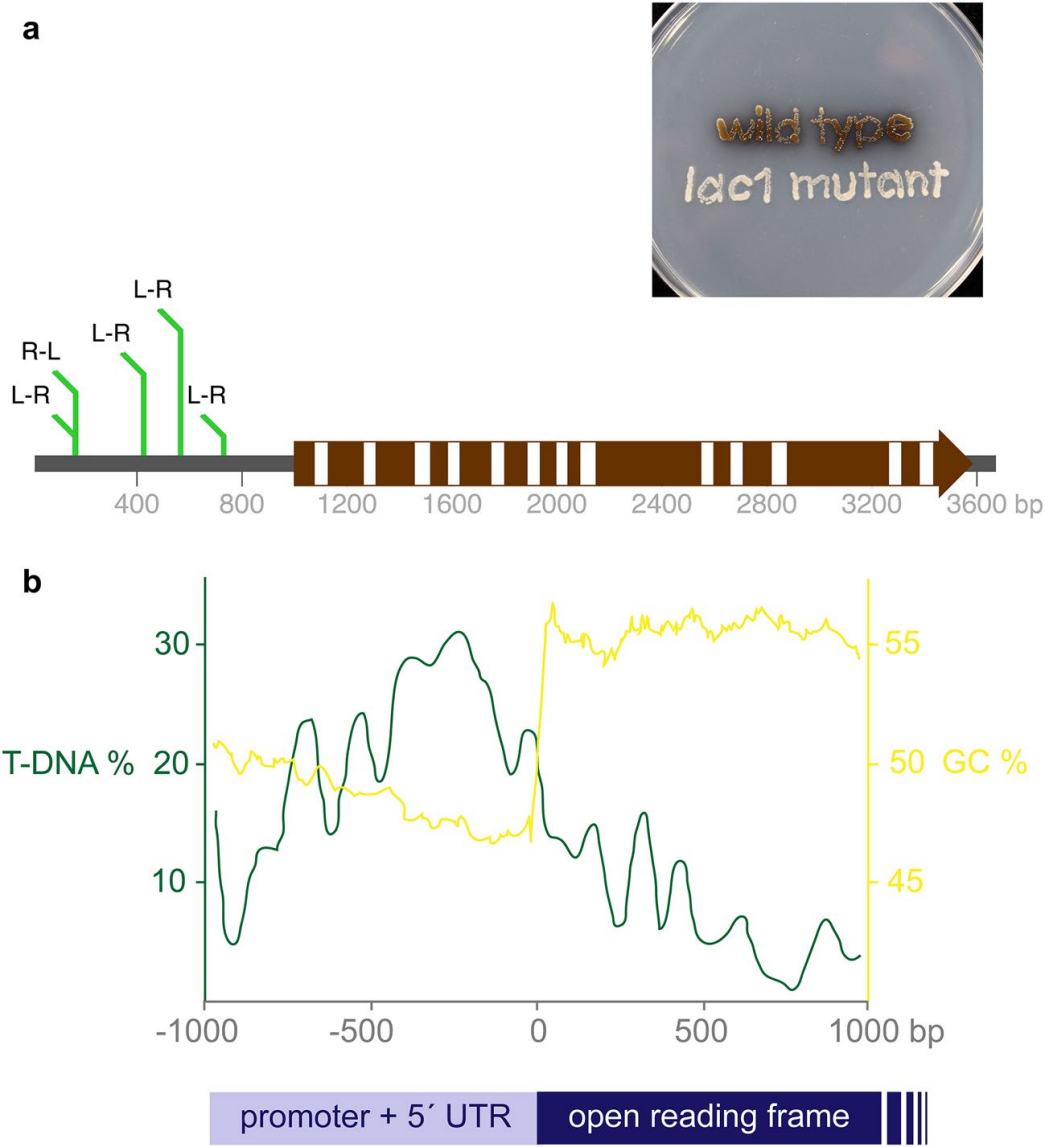
Two examples of T-DNA insertion bias into the 5′ non-coding (or flanking) regions of the genes in *Cryptococcus neoformans* (a basidiomycete yeast and human pathogen) and *Pyricularia oryzae* (a filamentous ascomycete and plant pathogen). **a** Example of a single gene in *C. neoformans* targeted by T-DNA insertion on five occasions [157, 159, 160] causing loss of pigmentation. A schematic drawing of the *LAC1* gene represented by an arrow shows coding regions as brown boxes, introns as white boxes and upstream and downstream non-coding sequences as a grey line. T-DNA insertions from five independent transformation events each causing loss of pigmentation all lie within the upstream region, with none to date in the coding region. R and L refer to the left and right border and the relative positions of these when the T-DNA inserted; note that the same site is targeted by two independent insertion events in opposite orientation. The plate shows *C. neoformans* wild type and one of the T-DNA insertional mutants growing on medium containing the substrate for laccase, l-DOPA. **b** Example of insertional bias of T-DNA into the genome of *P. oryzae* transformants. Results and figure are modified from Ref. [40]. An analysis of the distribution of 799 insertion sites mapped into 50 bp windows illustrates a twofold higher insertion frequency in promoter or untranslated regions compared to coding regions. This is even more striking in that such regions have a higher proportion of AT nucleotides while in *P. oryzae* T-DNA insertions are preferentially into higher GC content DNA

To gain insight about *C. neoformans* pathogenesis, T-DNA mutant screens have been performed using surrogate markers for virulence, such as changes in the production of the pathogenicity factors melanin and capsule, and the ability to grow at human body temperature (37 °C). Screens to identify the molecular basis of melanin biosynthesis exploit the presence of visible dark pigments produced by *C. neoformans* on media containing l-DOPA or other phenolic precursors on which melanin-deficient mutants are easily identified as white or pale colonies. This screen was performed as a proof-of-principle in the development of *C. neoformans At*MT with the identification of the genes *LAC1* and *CLC1*, encoding the main laccase involved in melanin biosynthesis and a putative voltage-gated chloride channel, respectively [157]. In a subsequent screen the same authors identified three more independent *lac1* mutants (Fig. 4a). Other mutants with melanin defects as well as mutants unable to grow at human body temperature were also identified [159, 160]. Successful identification of T-DNA mutants impaired in capsule production was performed by visual analysis of colony morphology or by selecting mutants unable to use heme, leading to the identification of the *CAP60*, *ARF1* and *VPS23* genes [89, 161].

Beside in vitro studies, a method to identify *C. neoformans* genes required for virulence in vivo used signature tags that were incorporated into the T-DNA molecules. Pools of signature-tagged T-DNA mutants were used for murine pulmonary infection experiments, and once the disease developed and the mice sacrificed, the reduction of the signature tag signal in the population of strains recovered from the mouse lungs indicated strains with possible reduction in virulence. This approach allowed the identification of a T-DNA mutant with reduced numbers of cells from the lungs. The impaired gene, *ENA1*, that encodes a putative ion transporter was then shown to be required for virulence in conventional virulence assays with an independently created gene deletion strain [160]. Remarkably, this application of *At*MT was the first forward genetic screen in a eukaryotic human pathogen that used virulence in animals as the phenotype. In contrast, such loss-of-virulence/pathogenicity screens have been extensively exploited with plant pathogenic fungi, as discussed above.

The application of *At*MT has also greatly contributed to decipher the mechanisms of light responses in *C. neoformans* and more broadly the fungi. While the *C. neoformans BWC1* gene encoding the blue light sensor was identified through searches of orthologs, other signaling components could not be identified through bioinformatic searches. Thus, a haploid strain was modified to become self-filamentous by the introduction of a construct to express the opposite mating type homedomain protein, and then used in a T-DNA insertional mutagenesis screen to find mutants with impaired sexual filamentation. One strain whose filamentation was not repressed when exposed to either light or dark had an insertion in the *BWC2* gene: the Bwc2 protein was then shown to directly interact with Bwc1 and be involved in the light responses during mating, and UV resistance. Furthermore, it was required for virulence [162]. The *BWC2* gene was independently identified by other investigators in subsequent *At*MT screens [163, 164], indicating a level of saturation in T-DNA mutant screens in this organism. Last, the downstream factor responsible for the UV sensitivity phenotype in *bwcl* or *bwc2* mutants was also identified as a UV sensitive T-DNA insertional mutant, in the gene encoding the Uve1 endonuclease required for repair of UV damage [165].

Using similar assays, *At*MT has been extensively used to identify genes required for mating and the transition from the yeast to hyphal form. A forward genetic screen performed on a hyperfilamentous strain derived from *Cryptococcus deneoformans* (previously *C. neformans* var. *neoformans*) isolated seven mutants unable to produce filaments during the α–α mating reproduction process (a homothallic mating system observed within the *Cryptococcus* clade). One mutant had a T-DNA insertion in the transcription factor gene *MAT2*, a key regulator of the sexual reproduction pathway essential for pheromone sensing, responding and cell fusion [166]. Two other independent screens using the same hyperfilamentous strain identified a new transcription factor, Znf3, that governs sexual reproduction though a *MAT2*-independent pathway, and Spo11 and Ubc5 proteins that are essential for sporulation [164], and the long non-coding RNA *RZE1*, which controls yeast-to-hypha transition through regulating the key morphogenesis regulator Znf2 [167]. Filamentation can still occur in the *mat2*Δ deletion mutant, and recently additional components required for filamentation were identified by a screen of 77,000 insertion mutants in a *mat2*Δ background [168]. *STE50* was identified as being required for all steps of monokaryotic fruiting and sexual reproduction, i.e. from response to pheromone to production of hyphae [169]. In these studies many other mutants with impaired mating and filamentation defects have been identified, and they include both previously characterized genes (e.g. *ZNF2, MAT2*, *STE7* and *BWC2*) and those of unknown function that at the present are subject to further studies.

*At*MT has also been used for new drug target discovery. In two studies *At*MT insertional mutagenesis was used to identify genes essential for viability. In the first it was performed on a diploid strain derived from *C. neoformans*, using Mendelian genetic segregation analysis on the haploid spores arising after meiosis [170]. In the second, a haploid strain was mutagenized by the introduction of a regulatable promoter within the T-DNA [16]. The essential genes identified are potential targets for new antifungal drugs.

Additional important discoveries in *C. neoformans* based on *At*MT include the identification of the *CTR2* gene required for evasion of macrophages [171], the RAM pathway components that confer altered colony morphology and control cell polarity [89], genes required for growth under hypoxic conditions [172], and the basidiomycetous-specific gene *RRA1* involved in the *RIM101*-mediated alkaline responses [173].

#### Pucciniomycotina

##### The Pucciniales that cause rust diseases

The rust-disease fungi represent the largest group within the Pucciniomycotina with about 7000 species that are obligate pathogens of many crop plants and trees, and for this reason they represent the most economically important group in this clade [174]. It is extremely difficult to conduct molecular studies and functional characterizations of genes in Pucciniales fungi for several reasons. First, as obligate biotrophic pathogens they cannot be cultured on artificial media; second, most of their life stages are dikaryotic, including the urediniospores that are commonly used in laboratory experiments; third, the need of a reliable transformation system that allows stable ectopic integration of the exogenous DNA; last, the lack of gene markers that force the selection of clear phenotypes only when the rust fungus is inoculated inside the host. Hence, the first and few reports on functional genetics consist of transient transgene expression achieved through biolistic transformation. However, transformation efficiencies were low and the transformants obtained were unstable, with two rare exceptions in *Puccinia triticina* [175].

The only successful and stable transformation experiment was in the flax rust fungus *Melamposora lini* using *At*MT [176]. The approach was exceptionally innovative. The genetic marker for selection was developed based on a previous finding that an avirulence gene of *M. lini* (*AvrL567*) when mutated can lead to disease in flax cultivars with a specific resistance gene (*L6*). Lawrence et al. used a hairpin antisense structure to silence the *AvrL567* avirulence gene and performed *At*MT of *M. lini* within the plant itself, i.e. within stems of a plant cultivar with no resistance genes that had been inoculated 5 days before with a *M. lini* isolate bearing homozygous copies of the *AvrL567* gene [176]. Candidate silenced urediospores were collected at different time points from these plants and inoculated into a cultivar containing the *L6* resistance gene, thus allowing the transformants to be selected by their ability to cause lesions. *M. lini* isolates that were able to cause disease were obtained, and molecular analyses confirmed stable integration of the T-DNA and robust silencing of the native *AvrL567* gene. Despite this elegant strategy, functional genetics through *A. tumefaciens* transformation-mediated gene silencing has not taken place yet in rust fungi and the work of Lawrence and colleagues is still the only report published [176].

##### *Microbotryum lychnidis-dioicae*

The Pucciniomycotina includes other plant pathogens, such as *M. lychnidis*-*dioicae* (*M. violaceum* sensu lato) that causes anther smut of plants in the Caryophyllaceae family. *M. lychnidis*-*dioicae* is a dimorphic non-obligate biotrophic fungus that has been intensively studied both at the genomics and genetics levels (see review [177]). It is considered a model system in non-agricultural settings and ecological studies, offering alternatives to study host–pathogen interactions in diverse host environments. Further, *M. lychnidis*-*dioicae* has been used as a model for studying the evolution of sex chromosomes in fungi, and it was the first fungus in which heteromorphic mating type chromosomes were described [178].

The potential of the resources that have been generated has not been fully exploited at the level of gene functions due to the lack of a reliable transformation system, despite early and apparently successful attempts. In 1989 Bej and Perlin reported the first transformation of *M. lychnidis*-*dioicae* [179] where they used lithium acetate and PEG to deliver into both protoplasts and intact cells a plasmid containing the *hygB* gene as the selective marker. Transformation efficiency was high and the exogenous DNA was integrated stably into the nuclear genome. Subsequently, the same authors reported the successful transformation of *M. lychnidis*-*dioicae* using bacterial DNA conferring resistance to neomycin [180]. Despite the positive outcome achieved, these techniques were not reproducible by other researchers, and transformation attempts using biolistics were unsuccessful [177].

By exploiting the newly acquired genomic and transcriptomic data for *M. lychnidis*-*dioicae*, a robust transformation system based on *At*MT has recently been developed [181]. The selection markers delivered through *At*MT consist of endogenous promoters of the most highly expressed genes under different phases of the fungal lifecycle, as assessed by previous transcriptomic data [182], fused with the *HYG2* gene alone or in combination with *eGFP.* Stable and random integration of the T-DNA in the *M. lychnidis*-*dioicae* genome was achieved, and also expression/over-expression of inserted genes, corroborating transcriptomic data. Although this is the only report of stable transformation in *M. lychnidis*-*dioicae,* the authors showed its potential as an insertional mutagen, thus opening a new field of functional genetics in this fungus. Currently further molecular tools, such as the overexpression of a heterologous marker using a native promoter as the driver and a targeted knockout system, are under development [177].

##### Red yeasts

The red yeasts in the Pucciniomycotina are a polyphyletic group that included the four genera *Sporobolomyces*, *Sporidiobolus*, *Rhodotorula* and *Rhodosporidium*, but in a recent reclassification most of the *Sporidiobolus* and *Rhodosporidium* teleomorphic species were grouped with their anamorphic counterparts *Sporobolomyces* and *Rhodotorula*, respectively, and a new genus (*Rhodosporidiobolus*) was created [183]. Compared to ascomycetous yeasts, whose importance in biotechnology has been known since ancient times for fermented beverages and food, basidiomycetous red yeasts have been relatively understudied in terms of their potential importance in biotechnology, agriculture, food processing, and environmental impact. The last few decades have revealed that these yeasts have a multitude of unique beneficial attributes, which include the production of secondary metabolites such as carotenoids and fragrances, sources of enzymes important in pharmaceutical production and chemical syntheses, biodegradation of pollutants and mycotoxins, antagonistic activity against plant pathogenic fungi, and high levels of lipid synthesis for biofuel production [184].

Gene functions in red yeasts were unexplored, with a single report of transformation in 1985 and nothing afterwards until 2010 [185]. In 1985 *Rhodosporidium toruloides* was transformed with a protoplast-lithium acetate/PEG approach: although transformation was successful, it yielded a very low number of transformants (~10^3^/µg DNA) and the majority of which were unable to retain the introduced exogenous DNA. Since 2010 there has been a drive to generate transgenic strains by two methods, biolistic and *Agrobacterium* T-DNA delivery.

Initial *At*MT attempts in red yeasts were unsuccessful due to the lack of Pucciniomycotina-specific gene markers and the capability of some species to become spontaneously resistant to the common drugs used in transformation protocols in the Agaricomycotina and Ustilagomycotina yeasts. The first successful *At*MT of red yeasts was for *Sporobolomyces* sp. IAM 13481 [186], whose draft genome sequence and annotation had been released by the Joint Genome Institute (JGI). The strategy employed was based on the use of the endogenous *Sporobolomyces* sp. IAM 13481 *URA5* and *URA3* genes as selective markers to restore prototrophy in *ura5* or *ura3* auxotrophs that were isolated as spontaneous mutants resistant to 5-fluoroorotic acid [186]. However, plasmids developed for *Sporobolomyces* were not effective for other Pucciniomycotina species, and to fill this gap several binary vectors were generated with other selective markers. These include the *URA5* and *URA3* genes of *Rhodotorula graminis* strain WP1, the naturally high G+C content gene encoding nourseothricin acetyltransferase placed under the control of the promoter and terminator of the tubulin-encoding *TUB2* gene [187], and a codon-optimized hygromycin phosphotransferase gene alone or fused with a codon-optimized enhanced green fluorescent protein gene, both placed under the control of endogenous promoter of the *GPD1* gene. These latter markers were used successfully to transform *R. toruloides* [188].

The crucial factors for successful transformation of red yeasts were selectable markers that were native copies of genes or including native regulatory elements, appropriate G+C content, or recoding the DNA for optimal expression [187, 188]. Following these key findings, vectors based on other antibiotic-resistance marker genes (bleomycin) and/or other promoters to drive gene expression were also evaluated. These experiments demonstrated multiple stable T-DNA integrations in the genome of *R. toruloides* [189], and showed that strongest heterologous expression was achieved using the constitutive glucose 6-phosphate isomerase promoter [190]. To date *At*MT is effective in transforming all the Pucciniomycotina red yeasts tested, which include species within the genera *Sporobolomyces* and *Rhodotorula*, and *Cystobasidium slooffiae* which belongs to a different class (i.e. Cystobasidiomycetes) [187]. The *At*MT tools have yielded new insights into genes required for biological processes in the Pucciniomycotina, as illustrated by the following examples.

A T-DNA forward genetic screen was developed with the aim to elucidate the genetic mechanisms behind the ability of red yeasts to resist and degrade the mycotoxin patulin, using *Sporobolomyces* sp. IAM 13481 as a model [191]. Although mutants defective in patulin degradation were not identified (chemical analysis subsequently revealed two independent pathways for degradation in this strain), genes involved in resistance to diverse stresses were identified. The putative functions of the mutated genes as well as the mutant phenotypes correlated with the cytotoxic effects of patulin, allowing the formulation of a hypothesis, which was then substantiated by transcriptomic analyses [192], that *Sporobolomyces* sp. IAM 13481 activates stress responsive genes to overcome the initial insult caused by the mycotoxin.

In another study, *At*MT insertional mutants of *Sporobolomyces* sp. IAM 13481 were used to investigate the molecular basis of the intriguing mechanism of ballistospore formation and dispersal that is unique to basidiomycete fungi. This property can be evaluated in the laboratory by the production of a mirror image of a culture from one Petri dish onto an uninoculated second dish [193]. The screen of more than 5000 transformants led to identification of 18 *mirror* mutants unable to shoot spores to produce the mirror image. One mutant bearing a T-DNA insertion in gene *PHS1*, which encodes 3-hydroxyacyl-CoA dehydratase required for the third step in very long chain fatty acid (VLCFA) biosynthesis, was further subjected to molecular, phenotypic and biochemical characterizations that unequivocally confirmed the involvement of Phs1 and VLCFAs in basidiomycete dispersal through a delay in ballistospore formation. Strikingly, *PHS1* is an essential gene in other fungi, and its identification in a *Sporobolomyces* sp. mutant was fortuitous as the T-DNA inserted within the last intron of the gene and past the essential amino acid residues required for function, which resulted in the production of an altered transcript yet still viable phenotype [193].

An industrial and biotechnological application of gene manipulation by *At*MT has been recently reported for *R. toruloides* [194]. In order to achieve a higher rate of lipid production, *At*MT was used to generate metabolically engineered strains through a “push–pull” approach for two genes involved in triacylglycerol biosynthesis. A plasmid for *At*MT was manipulated to overexpress simultaneously the *ACC1* and the *DGA1* genes, which encode an acetyl-CoA carboxylase and a diacylglycerol acyltransferase, with the glyceraldehyde-3-phosphate dehydrogenase and the ATP-citrate lyase promoters, respectively. Due to the random integration of the T-DNA into the genome, the transformants showed variability in the production of lipids. However, one transformant had a 2-fold increase of lipids during growth on glucose, compared to wild type.

Beside the random integration of the T-DNA into the host genome, *At*MT also can generate targeted gene replacements through homologous recombination in the Pucciniomycotina. This was first demonstrated using a strategy that exploited the change in pigmentation of *Sporobolomyces* sp. IAM 13481 from red to white when mutating the *CAR2* gene [187]. Based on the proportion of white transformants obtained, at least 1000 bp of flanking DNA is necessary to achieve a good percentage (~6%) of homologous recombination events. This percentage was about half of that achieved using biolistics transformation, which was included as a comparison since it was known to be effective for target gene replacement in *Sporobolomyces* sp. IAM 13481 [186, 187]. However, one advantage of *At*MT compared to biolistic transformation is that it does not require expensive equipment but the basic resources that are common in a molecular biology or microbiology laboratory. A *ku70*∆ mutant of *R. toruloides* was generated that showed a drastic improvement of target gene replacement efficiency through homologous recombination, with values ranging from 20% of deletion mutants obtained using just 100-bp of flanking regions to ~91% when 1000 bp or 1500 bp were used [195].

A last point worth raising on the role of *At*MT in the Pucciniomycotina is that Liu and colleagues used the reliability of the *At*MT of *R. toruloides* to develop an inducible promoter system for red yeasts [196]. The *DAO1* gene promoter was fused to a codon-optimized luciferase gene, which was then inserted by *At*MT at the *CAR2* locus of the *R. toruloides ku70*∆ strain, allowing the monitoring of the luciferase expression driven by the *pDAO1* in the albino mutants. An optimized *DAO1* promoter, which contains an intronic enhancer sequence and an artificially created ATG start codon, tightly induces luciferase expression in the presence of d-alanine; conversely, its expression was repressed by l-alanine or glucose and ammonium sulfate. More recently, to further exploit the biotecnological potential of *R. toruloides*, the same authors characterized the promoters of six genes involved in lipid biosynthesis, and identified that the promoter of the perilipin/lipid droplet protein 1 gene (*LDP1*) displays 4- to 11-fold stronger activity than that of the glyceraldehyde-3-phosphate dehydrogenase gene (*GPD1*), one of the strongest promoters known in yeasts [197].

#### Ustilaginomycotina

The third subphylum in the Basidiomycota with a large number of species is the Ustilaginomycotina. These are best known for the species that cause smut diseases of plants. Deployment of *At*MT in these basidiomycetes has been patchy. For *U. maydis* and close relatives, where protoplast-based methods have been routine, and gene disruption was already easy, there was no immediate advantage in deploying *At*MT. However Ji et al. [198] used *At*MT in *U. maydis* for efficient transformation and gene disruption including on previously frozen cells and obviating any need for protoplasting, and Ali et al. [20] used *At*MT to deliver large-insert vectors into *U. hordei*. *At*MT has also been used to transform *Pseudozyma antarctica* (*Moesziomyces antarcticus*), which is an extremophile due to its growth under low temperatures and hence of interest for the potential cold-adapted enzymes it may produce [199].

Recently, *At*MT was found to be the only transformation method effective in species of *Malassezia* [200, 201]. This genus is associated with skin and hair of animals, and represents the main fungal component of the human skin microflora [202]. Despite this high prevalence and links to common human skin diseases, the genus had been poorly studied at the level of gene functions in part due to challenges in culturing the species, all of which are fatty acid auxotrophs. Building on recently developed genomic resources [203], *At*MT was optimized for *M. furfur*, *M. sympodialis* and *M. pachydermatis*, with co-cultivation conditions modified to support higher levels of transformation. A noteworthy aspect of these experiments was high frequencies of targeted gene disruptions (>60% of transformants being knockouts) for two genes in *M. furfur* [200].

## Fungi outside the Dikarya

The Ascomycetes and Basidiomycetes form a monophyletic lineage in the fungi (Fig. 2), termed the Dikarya due to the presence of two-nucleus cells during the sexual stages of their lifecycle. However, there are at least nine additional lineages that at one point were classified into two groups, the zygomycetes and chytrids, many with limited molecular biology. Given the promiscuity of *A. tumefaciens*—it can transform plants, fungi, oomycetes and human cells [23, 24]—one application of *At*MT that is relatively limited is in the fungal species outside of the Dikarya lineage (Fig. 2).

The Mucoromycota are a large group of species and gene function has been studied through isolation of mutants by chemical mutagenesis screens, and through targeted gene disruption in a few species [204]. *At*MT has been applied to a number of these species, and it was first reported in the Mucoromycotina species *Rhizopus oryzae* [205]. A common limitation is the loss of the transgenes in species such as *Backusella lamprospora* or *Mucor* spp. [206–209], which may reflect transient transformation, gene silencing or a foreign DNA surveillance system, or loss over time during passaging of these species that have coenocytic hyphae (without septa) and multinucleate spores. *Umbelopsis isabellina* is an interesting case, wherein standard *A. tumefaciens* strains used to transform fungi were compared with a strain of *A. rhizogenes*, which is another *Agrobacterium* species able to transform plants [210]. In this case, the latter bacterial species yielded higher numbers of transformants.

To date, the most successful applications of *At*MT in a biotechnology capacity have been on the Mortierellomycotina subphylum, in the species *Mortierella alpina*, which is a source of polyunsaturated fatty acids that may be beneficial for health. Some fatty acids derived from *M. alpina* are added to infant formula, while others like eicosapentaenoic acid are only otherwise available from fish oil. The initial transformation approaches aimed to modify or increase the fatty acid composition of this fungus [211, 212]. Subsequent studies altered gene expression to change the lipid profiles by overexpressing an ω3-desaturase enzyme or pathways that alter NADPH levels [213–215].

Other than the Mucoromycota, a chytrid species in the Blastocladiomycota, *Blastocladiella emersonii*, has been reported as transformed using *Agrobacterium* [216]. The Glomeromycota are obligate symbionts with the roots of plant species, and are challenging to work with for this reason. For instance, use of drugs for selection would likely also impede growth of the host plant. *Rhizophagus irregularis* (previously *Glomus intraradices*) has also been subjected to *At*MT, using as a selection system the delivery of constructs that express a nuclear-localized GFP [217]. The method was inefficient, hindered by the natural levels of autofluoresence of the fungus, and transformation success could not be confirmed with other approaches.

## All that shines is not silver: problems and limitations with the *At*MT technique

The previous sections are highlights in which the use of *At*MT has led to new and major advances in fungi. However, not all is perfect with the tool, and researchers should consider some of these limitations when designing forward genetic experiments, planning to make targeted gene replacements, or interpreting data from strains generated with *At*MT.

Given that generating hundreds or thousands of mutants is a prerequisite for functional genomic studies, several adaptations to the *At*MT pipeline have been made to overcome significant experimental limitations and ensure this technique is sufficiently high-throughput. In some instances, technical challenges cannot be obviated. For example, while 96-well high throughput protocols exist for lithium acetate or protoplast-PEG mediated transformation of model yeasts [218], similar experiments in 96-well format are not possible using *At*MT. This is likely due to the technical challenges of miniaturising *Agrobacterium* cultures, which must be sufficiently aerated to reach an optimised growth phase prior to transformation. Nevertheless, much progress has been made in reducing the burden of experimentally intensive cloning for *At*MT, which relies on generation of *Agrobacterium* compatible plasmids. Such cloning is more challenging when compared to lithium acetate or PEG mediated transformation, where linear DNA cassettes can be assembled by simple PCR steps [219]. Investigators have generated *Agrobacterium* plasmids that are compatible with Gateway^®^ cloning technology from Invitrogen, e.g. enabling ultra-high throughput generation of *Z. tritici* over-expression strains [220]. Elsewhere, *Agrobacterium* compatible vector construction using yeast recombination in *S. cerevisiae* [14–16], or Golden Gate assembly [18], provide comparable improvements in high throughput vector construction. These studies highlight how some important technical challenges have been addressed, and their recent utilization demonstrate *At*MT is applicable for high throughput functional genomic analyses of fungi [220]. More generally, they highlight that *At*MT is a robust technique that will continue to be essential for analysis of fungi in the “big data” era of fungal functional genomics, as long as *At*MT is used taking into consideration both the advantages and the limitations of the technique. Four points to consider are as follows.

First, an ideal insertional mutagenesis protocol should provide the ability to “hit” any and every gene, but T-DNA insertions have non-random integration patterns into fungal genomes. A single gene example of this bias is illustrated by the isolation of T-DNA insertional mutants into the *LAC1* gene encoding laccase for melanin biosynthesis in *C. neoformans*. Five insertions have been isolated within a 1 kb promoter region and none in the 2.8 kb coding region [157, 159, 160] (Fig. 4a). This single gene example is consistent with insertional bias from the analysis of collectively thousands of T-DNA insertion strains in the plant pathogenic ascomycetes (see examples given above, and illustrated from *P. oryzae* in Fig. 4b). This is perhaps the biggest limitation to the use of *At*MT as a tool for creating a library of mutants with insertions in every gene.

Despite this limitation, the skew to insert the T-DNA outside of genes has been recently converted into an advantage for the identification of essential genes [16]. The regulatory sequences for the *C. neoformans GAL7* gene were cloned adjacent to the right border, and transformed into the basidiomycete *C. neoformans*. Transformation and selection was conducted on media containing galactose to ensure any gene near that insertion would be expressed, and then strains tested for growth on glucose, which represses *GAL7* expression. Approximately 1% of transformants did not grow on glucose media. Analysis of the positions of the T-DNA insertions in the *C. neoformans* genome revealed they were in genes with essential functions in ascomycete species.

Second, in ideal cases the T-DNA inserts perfectly from right to left border, the subsequent mutant (or set of mutants) with an interesting phenotype is isolated, followed by rapid identification of the gene of interest. Identification of the deleted or disrupted gene might be achieved by TAIL-PCR, inverse PCR, or some other method. However, this gene identification step can become a bottleneck or even end point for several reasons. The T-DNA may insert in ways which make subsequent mapping challenging, for example following signficiant cassette truncation, insertion of additional plasmid DNA beyond the border sequences, or with multiple copies either inserted in tandem or dispersed throughout the genome. The insertion events can be associated with deletions and chromosomal rearrangements of the genome. The tendency for insertions to fall within intergenic regions can cause issues establishing which of the two genes may be affected by the T-DNA, and sometimes qPCR of both genes can help identifying which one is affected by the insertion. In many cases the assumption is that the insertion will reduce expression of the adjacent genes. However, there are also examples such as from *L. maculans* in which the insertion event causes an increase in gene expression of adjacent genes [95].

Third, *At*MT may not be able to provide valuable insighs compared to other transformation methods in some species. For a number of fungi, *At*MT did not enhance our understanding of gene function in fungi. This includes the first fungus transformed using *A. tumefaciens*, *S. cerevisiae* [2]. This makes sense given the other transformation techniques available for *S. cerevisiae*, the ability to use chemical mutagenesis and then clone by complementation, construction and access to sets of strains with all the genes deleted, and other genomic-level resources. Hence, with so many tools to isolate mutants and identify the affected gene, or screening whole genome deletion sets for phenotypes, there was little additional benefit of having *At*MT as another tool. As mentioned above, a similar situation exists for the filamentous fungus model *N. crassa*: although one of the first filamentous species transformed with *Agrobacterium* [4] the method was not used since that time. A third example is *Schizosaccharomyces pombe*, a model used to uncover regulators of cell cycle control that was the topic of the 2001 Nobel Prize in Physiology or Medicine; indeed, this species and it seems no member of the Taphrinomycotina have been transformed with this method.

A fourth limitation with *At*MT is that transformation with T-DNA will always be insertional, whereas in some fungi, such as *U. maydis* [221] or with the use of the AMA1 sequence in plasmids for *Aspergillus* spp. [222], plasmids that replicate autonomously are available. Such plasmids can be rescued back into *E. coli*, easing the way for cloning by complementation, or for deliberate loss by counter-selection.

## The future and further maximizing the impact of *At*MT in fungi

*At*MT is a tool that is applicable to plant and fungal molecular biology, but the host side of the transformation is little understood. A set of 129 *A. thaliana* mutants has been identified that are resistant to transformation [223]. However, the fungi surpass all other eukaryotes in terms of the molecular tools and resources available for their study at the genome-wide scale and as such could be ideal hosts to define the eukaryotic side of the host cell–bacterial cell interaction. The process of T-DNA transfer and integration has been recently reviewed [7]. Using fungi as the host could provide insights into ways of improving efficiency of the method and the mechanisms by which the T-DNA is integrated. *S. cerevisiae* mutants have been used to identify those that have altered efficiency of transformation [224–229], and a discovery of the role of purine concentrations and biosynthesis was then also linked to transformation efficiency in plants [230]. Hence, testing other deletion sets, such as for *S. pombe* or the ongoing projects for *N. crassa* or *C. neoformans*, to find strains recalcitrant to transformation. The rationale for exploring the eukaryotic genes required for transformation in multiple species is because there are clear differences between fungi, e.g. gene replacement using *At*MT is not possible in *C. neoformans* but it is in other basidiomycetes or in *S. cerevisiae* the influence of purine during transformation on efficiency is dependent on both genes for purine synthesis and the yeast strain [230]. This research direction could have wide impact, if that information could be transferred across to explain why some plant species are difficult to transform with the method. The other side of the transformation interaction is *A. tumefaciens* itself. There is evidence that different strains of *A. tumefaciens* behave differently. Few studies have investigated strains side-by-side for bias in insert preference or gene targeting efficiency.

*At*MT can be a powerful teaching tool. “It is in human nature to value any novelty, however slight, in one’s own possession” [231]. Those who have discovered a new mutant or mutant phenotype will appreciate the insight of Charles Darwin’s comment, particularly for engaging students or others at many education levels with science. It is not known how many people have used *At*MT as a teaching tool on the philosophy underlying molecular genetics, i.e. the phenotype of a mutant reflects the inverse of its function in the cell. In an undergraduate practical class setting, it is an effective method for demonstrating this principle, as well as exposing students to fundamental aspects of biology. The method has been taught at the Marine Biological Laboratory Molecular Mycology course (Woods Hole, MA, USA) since 2004 where students have performed large-scale mutant screens as part of research projects [166, 173, 232].

Several of the authors of this review have used *At*MT as a teaching tool for undergraduates or even high school students, in cases resulting in the students contributing to research publications [89, 159, 193, 233]. For implementing *At*MT as a teaching tool, it is wise to select phenotype screens whereby multiple genes can control that particular phenotype to increase the chances of students isolating mutants.

It is essential to make any new method or resources available, such as through the deposition of plasmids or strains. One drawback of the use of *A. tumefaciens* is that some countries classify laboratory strains of *A. tumefaciens* as plant pathogens and have import restrictions due to this classification. An invaluable role has been and continues to be played by public repositories, such as the Fungal Genetics Stock Center in the USA. However, lack of funding for such organizations both threatens these and undermines decades of research and limits the potential to perform experiments into the future [234]. In addition to the physical resources, online databases of genomes, T-DNA insertions and phenotypes (Fig. 3) also require ongoing support to avoid the loss of this hard-gained information.

At present, the identification of T-DNA junctions can still be a rate-limiting step. Sequencing a genome can be more cost effective than PCRs or other methods to identify junctions. Indeed, next generation sequencing has been applied for the identification of the mutated genes. In the case of *L. maculans*, there was difficulty in identifying junctions and four strains were sequenced separately to identify the T-DNA insertion sites [100]. Another approach was used for *C. neoformans* in which pools of DNA from mutants were sequenced simultaneously, and then the affected genes in the individual strains identified by specific PCRs [168, 232]. In future, incorporating signature tags, bar codes or using asymmetric restriction enzymes will make identification of flanking regions easier with the next-generation sequencing capabilities that are available.

In plants there have been many reports of using co-transformation to introduce multiple T-DNA cassettes at the same time (e.g. [235]); however, this is not a common approach in fungi [236]. Such an approach has the potential to allow *At*MT delivery of separate partial selection cassettes, in a similar approach to that used very successfully in the bipartite or split-marker approach to enhancing targeted gene disruption in fungi [219]. Future implementation may further enhance gene targeting abilities in fungi [237, 238].

An exciting tool in biological research is the CRISPR/Cas genome editing systems and likely, as *At*MT did in the last 2 decades, this methodology could revolutionize fungal biology. As with *At*MT in *S. cerevisiae*, it is likely that for the well-established fungi CRISPR/Cas will be less ground breaking than for those species with challenges, especially species with low frequencies of targeted gene replacements. It is also worth pointing out that for many fungi *At*MT will still be an instrumental tool to transform in the CRISPR/Cas constructs. Another feature of *At*MT yet to be exploited in fungi is transient transformation. In plants this has been a routine method for introduction of constructs such as viral infectious clones [239], and given the difficulty currently faced with manipulation of mycoviruses, it is likely that such deployment will occur in the future. There is also interest in exploiting the transient transformation systems for other purposes like CRISPR/Cas that would circumvent the need to have this system integrated into the genome and the potential that causes for off-target mutations. In Ascomycetes this has been achieved by exploiting unstable autonomous vectors delivered by protoplast transformation [240, 241], whilst in *U. maydis* an unstable autonomous plasmid was successfully deployed into protoplasts to deliver an efficient CRISPR/Cas system [242], but clearly there is also the possibility of exploiting the benefits of *At*MT for such cases in the future, potentially by modification of some of the components required for integration of the T-DNA into the fungal genome.

## Concluding remarks

In closing, nearly 20 years on we have a chance to answer Dunn-Coleman and Wang’s question about *At*MT, “a silver bullet for filamentous fungi?” This “silver bullet” conjures a mixture of concepts, ranging from the use of silver against werewolves, Paul Ehrlich’s “magische Kugel”, or the lesser metal status of silver over gold. The perfect agent to comprehensively determine gene function in fungi, which ultimately provide these species with their remarkable capabilities, is likely elusive. *At*MT has, however, achieved many milestones in defining the genetic basic behind numerous and diverse traits, and promises to continue to play an important role for our understanding of fungal biology.
